# Human alveolar epithelial type II cells in primary culture

**DOI:** 10.14814/phy2.12288

**Published:** 2015-02-13

**Authors:** Pu Mao, Songling Wu, Jianchun Li, Wei Fu, Weiqun He, Xiaoqing Liu, Arthur S Slutsky, Haibo Zhang, Yimin Li

**Affiliations:** 1 State Key Laboratory of Respiratory Diseases and Guangzhou Institute of Respiratory DiseasesGuangzhou, Guangdong, China; 2 The First Affiliated Hospital of Guangzhou Medical UniversityGuangzhou, Guangdong, China; 3 Keenan Research Centre for Biomedical Science of St. Michael's HospitalToronto, Ontario, Canada; 4 Department of Medicine, University of TorontoToronto, Ontario, Canada; 5 Department of Anesthesia, University of TorontoToronto, Ontario, Canada; 6 Department of Physiology, University of TorontoToronto, Ontario, Canada

**Keywords:** Human lung, lamellar bodies, surfactant proteins

## Abstract

Alveolar epithelial type II (AEII) cells are a key structure and defender in the lung but also are the targets in many lung diseases, including acute respiratory distress syndrome, ventilator-induced lung injury, and pulmonary fibrosis. We sought to establish an optimized method for high yielding and long maintenance of characteristics of primary human AEII cells to facilitate the investigation of the mechanisms of lung diseases at the cellular and molecular levels. Adult human peripheral normal lung tissues of oncologic patients undergoing lung resection were collected. The AEII cells were isolated and identified by the expression of pro-surfactant protein (SP)C, epithelial sodium channel (*α*ENaC) and cytokeratin (CK)-8, the lamellar bodies specific for AEII cells, and confirmed by the histology using electron microscopy. The phenotype of AEII cells was characterized by the expression of surfactant proteins (SP-A, SP-B, SP-C, SP-D), CK-8, KL-6, *α*ENaC, and aquaporin (AQP)-3, which was maintained over 20 days. The biological activity of the primary human AEII cells producing SP-C, cytokines, and intercellular adhesion molecule-1 was vigorous in response to stimulation with tumor necrosis factor-*α*. We have modified previous methods and optimized a method for isolation of high purity and long maintenance of the human AEII cell phenotype in primary culture. This method provides an important tool for studies aiming at elucidating the molecular mechanisms of lung diseases exclusively in AEII cells.

## Introduction

Lung alveolar epithelium is composed of type I and type II cells (Mason 2006). Alveolar epithelial type II (AEII) cells are cuboidal and constitute 60% of all alveolar epithelial cells (Crapo et al. 1982). AEII cells play a key role in maintaining the integral function of the pulmonary alveolus (Driscoll et al. 1995), and serve as progenitors of AEI cells contributing to alveolar epithelial repair (Uhal 1997; Fehrenbach 2001). AEII cells are considered as a defender of the alveolus given their physiological and biological functions to synthesize, secrete, and recycle all components of the surfactant that controls alveolar surface tension (Mason and Williams 1977). AEII cells regulate the transepithelial sodium ion transport for alveolar fluid clearance, modulate the balance of coagulation and fibrinolysis, and thus contribute to the host defense system. AEII cells not only guide neutrophils into the alveolar space during their emigration (Burns et al. 2003) but also act as effector cells through interaction with resident and mobile cells, either directly by cell–cell contact or indirectly by ligand–receptor crosstalk through releasing cytokines and growth factors. AEII cells are particularly vulnerable to mechanical stress, as evidenced in patients with acute respiratory distress syndrome (ARDS) and in the context of ventilator-induced lung injury, and in ex vivo model of mechanical stretch systems of lung epithelial cells (Dreyfuss et al. 1988; Hotchkiss et al. 2002; Cabrera-Benitez et al. 2012; Huang et al. 2012).

To understand the mechanisms of ARDS and ventilator-induced lung injury at cellular and molecular levels, most investigations focusing on alveolar epithelial mechanotransduction, repair and phenotype transition have been conducted using human bronchial epithelial cells (i.e., BEAS-2B), AEII-like cell lines (i.e., A549), or primary AEII cells from rodents (Sanchez-Esteban et al. 2001; Oudin and Pugin 2002; Desai et al. 2008). Several increasing concerns have arisen about the differences of activation of signal pathways seen between bronchial and alveolar epithelial cells, the loss of human AEII cell phenotype in the transformed cell lines and the different functions of AEII cells between human and animals (Kreda et al. 2001).

Several groups have developed methods to isolate rat and human AEII cells in primary culture (Dobbs 1990; Cunningham et al. 1994; Corti et al. 1996; Wang et al. 2007, 2011; Ballard et al. 2010; Lazrak et al. 2012). Under most culture conditions, primary AEII cells tended to differentiate into AEI cells and it has to date proven difficult to maintain surfactant-producing human AEII cells in culture.

To overcome the problems mentioned above, we have combined and modified previous techniques (Ehrhardt et al. 2005; Yu et al. 2007; Bove et al. 2010) to isolate human AEII cells from the peripheral portion of tumor-free lung tissue of patients undergoing lung resection. Our approach was focused on the improvement and the maintenance of AEII cell phenotype and the cell function in this study.

## Methods

The study was approved by the Institutional Research Ethical Board of the First Affiliated Hospital of Guangzhou Medical University (201232) for the use of human material isolation of primary AEII cells.

### Specimens and isolation

The method of isolation was established by adaption and modification of several previous protocols (Ehrhardt et al. 2005; Yu et al. 2007; Bove et al. 2010) (Table1). Under sterile conditions, the specimen of distal portions of normal lung tissue (6–10 g) was obtained from patients undergoing lung resection. The specimen was chopped into 1 cm^3^ in size and washed with HEPES-buffered balanced salt solution (BSS) until clear. The lung pieces were minced (0.5 mm^3^) with eye scissors and then transferred into a sterilized beaker containing 50 mL BSS. After gentle mix, the solution of tissue was filtered passing through a mesh (150 *μ*m) cell strainer (BD Falcon, San Jose, CA). The remaining tissue was washed at least three times, and incubated in a solution containing 3 mL of trypsin (10,000 U/mL; Sigma, Saint Louis, MO) and 300 *μ*L of elastase (5.1 U/mL; Worthington, Lakerwood, NJ) for 45 min in a shaking water bath at 37°C. DNase I at 2 mL (10,000 U/mL, Sigma, Saint Louis, MO) for 15 min. The enzymatic activity was stopped using 40 mL inhibition solution (30 mL DMEM/F-12, 1:1, Invitrogen; Grand Island, NY 10 mL FBS and 1 mL DNase I 10,000 U/mL).

**Table 1:** Comparison of various protocols used for isolation of human AEII cells

Protocol	Current study	Reference Yu et al. (2007)	Reference Ehrhardt et al. (2005)	Reference Bove et al. (2010)
Tissue	Distal portions of normal lung tissue of lung cancer undergoing lung resection	Cadaveric lung declined by donor network	Distal portions of normal lung tissue of lung cancer undergoing lung resection	Normal lung rejected for transplant
Perfusion	Not applied	Pulmonary artery perfusion	Not applied	Main stem bronchus lavage
Digestion buffer	Use of trypsin (10,000 U/mL) to mince lung tissue, and elastase (5 U/mL) for digestion	Use of 13 U/mL elastase for digestion	Use of trypsin (10,000 U/mL) to mince lung tissue, and elastase (60 U/mL) for digestion	Use of 13 U/mL elastase for digestion
Filtration	150, 75, and 40 *μ*m in tandem	150, 30 *μ*m in tandem	40 *μ*m	150, 40 *μ*m in tandem
Fibroblast removal	Adhesion medium, 150 min 3 times	NO	Adhesion medium, 90 min, once, and anti-fibroblast magnetic beads	NO
Macrophage removal	Adhesion medium Anti-CD14 magnetic beads	Anti-CD14 magnetic beads	Adhesion medium Anti-CD14	IgG antibody-coated dishes
Culture medium	SAGM+1%FBS	MEM+10%FBS+2%high growth factor matri-gel	SAGM	DMEM+10%FBS
Morphology	Alveolar-like cysts	Alveolar-like cysts	Alveolar epithelial type I-like	Cobblestone
Cell markers	Lamellar bodies, SP-A, SP-B, SP-C, SP-D; Cytokaretin -8, KL-6, AQP-3	Papanicolaous lamellar bodies, proSP-C	Papanicolaous	Lamellar bodies, proSP-C
Time points of AEII phenotype Evaluation	12, 21, 28 days	4 days	6–10 days	5 days

The digested suspension was then diluted by adding 400 mL BSS and thoroughly whiffled for 10 min with a Pasteur pipet. The suspension was filtered through cell strainers at the size of 150, 75, and 40 *μ*m in tandem to collect the crude cell suspension. After centrifugation at 400 g for 10 min at room air, the cell pellet was resuspended in adhesion medium for macrophages and fibroblasts (22.5 mL DMEM/F-12, 1:1; 22.5 mL small airway epithelial cell growth medium, SAGM, Lonza, Walkersville, MD; 5 mL FBS and 1 mL DNase I 10,000 U/mL) and transferred to 100 mm Petri dishes (10 mL each). After incubation at 37°C for 150 min, an optimized time from our pilot studies, macrophages and fibroblasts attached on the cell culture dishes were separated from epithelial cells which took longer time to be attached in the medium (Dobbs 1990; Yamaya et al. 2002; Wu 2005). This differential adhesion procedure was repeated three times, and then the AEII cells were gently collected and centrifuged at 300 g for 10 min at room air. The cell pellets were resuspended in 3 mL DME/F12 and the crude cell suspension was layered on a 1.040–1.089 g/mL discontinuous Percoll gradients. A solution of 40 mL light gradient (1.040 g/mL) contained 4 mL PBS 10×, 12.55 mL Percoll solution (MP Biomedicals, Solon, OH), and 23.45 mL distilled water. A solution of 40 mL heavy gradient (1.089 g/mL) contained 4 mL PBS 10×, 25.96 mL Percoll® solution (MP Biomedicals), 10.04 mL distilled water, and one drop of phenol red. Each solution at 3 mL of volume was gently loaded into FBS of 15-mL centrifuge tubes, followed by centrifugation at 300 g for 20 min at 4°C using a swing out rotor to generate an enriched layer of AEII cells at the interface between the two layers of Percoll gradients. The deceleration was achieved by turning off the power when the desired centrifugation time was reached. The enriched cell pellets were transferred to a 15-mL centrifuge tube containing 13 mL BSS and centrifuged twice at 300 g for 10 min at room air. The cells pellets were resuspended in 2 mL BSS and a magnetic bead separation approach was applied to remove any possible contamination of macrophages (Anti-CD14 MicroBeads; Miltenyi Biotec, Bergisch Gladbach, Germany). The isolated AEII cells were centrifuged at 300 g for 10 min at room air, resuspended in 2 mL SAGM containing 1% FBS, and incubated in 60-mm culture dishes at 37°C for 60 h while medium was changed every other day. The cells were then subcultured in either SAGM or DMEM containing either 1% or 10% FBS for up to 21 days.

### Cell viability and proliferation assays

The AEII cells (1 × 10^4^/mL) were plated in 24-well plates in 5% CO_2_ incubator at 37°C. The additional AEII cells (3 × 10^4^) were seeded onto cell culture inserts of six-well transwell system (0.4 *μ*m pore, Lowell, MA) with culture medium in both the apical and basal compartments. Upon confluence, the cells on apical surface exposed to air by removal of the culture medium from the apical compartment to form an air–liquid interface culture (Chen et al. 2009). Cell viability and counting were conducted at days 2, 21, and 28 using trypan blue exclusion assay. The population doubling level was defined as the total number of times the cell growth has doubled since the initial seeding after isolation, and was calculated with a formula (Hayflick 1973).

### Transmission electron microscopy

Transmission electron microscopy is the gold standard to identify AEII cells (Fraslon et al. 1993), as the technique can demonstrate clearly specific ultrastructural features such as lamellar bodies and microvilli. AEII cells were gently scraped and then fixed dehydrated, and embedded as published previously (Driscoll et al. 1995). Thin sections were cut, stained with uranyl acetate and lead citrate, and examined on a transmission electron microscope (Philips CM-10) operated at 80 KV.

### Evaluation of tight junction

To examine the tight junction, AEII cells (1 × 10^5^/well) were grown on electrodes standard 8-well array (8W10E+ Applied Biophysics, Troy, NY). The electric cell-substrate impedance sensing (ECIS) technique (Applied Biophysics, Troy, NY) was used to examine the activities of cell grown in tissue culture. The permeability of the monolayer AEII cells was assessed with a record of transepithelial resistance (TER) across the electrodes.

### DND-26 staining of lamellar bodies

LysoTracker® Green DND-26 is a fluorescent dye that accumulates preferentially in lamellar bodies in human primary AEII cells (Haller et al. 1998). The cells were cultured for 21 days and seeded on coverslips overnight, then incubated for 30 min with LysoTracker® Green DND-26 (150 nmol/L; Molecular Probes, Eugene, OR) at 37°C in SAGM containing 1% FBS. The coverslips were washed with BSS (prewarmed to 25°C) and mounted for confocal scanning analysis.

### Characterization of AEII cell phenotype by mRNA expression

Expression of genes including surfactant protein A (SPA), SPB, SPC, SPD, CK-8, keratin-6 (KL-6), AQP-3, but AQP-5, was detected as described previously (Zhang, Zhang et al. 2013). The sequences of primers are listed in Table2.

**Table 2:** Sequences of primers

Genes	Primer sequences(5′–3′)				Product length	Traditional RT-PCR/q-PCR
SP-C	Sense	CTCATCGTCGTGGTGATTGTG	Anti-sense	TGGAGAAGGTGGCAGTGGT	154 bp	Both
SP-A	Sense	CGACTTTAGACATCAAATCCTGC	Anti-sense	CTCGGTACCAGTTGGTGTAGTTT	305 bp	Traditional–PCR
SP-A	Sense	CAGGTAGTGTTCCAGCAGGGTG	Anti-sense	ATCTGAAGGCGGCTCTAGGTCA	149 bp	q-PCR
SP-B	Sense	AGAGCAGGAGCCAGGGATGT	Anti-sense	AGCAGGATGACGGAGTAGCG	324 bp	Traditional–PCR
SP-B	Sense	CCAAGCCATGATTCCCAAGGGTG	Anti-sense	CGAGCAGGATGACGGAGTAGCG	122 bp	q-PCR
SP-D	Sense	AGGAGCAAAGGGAGAAAGTGG	Anti-sense	GCTGTGCCTCCGTAAATGGT	197 bp	Both
KL-6	Sense	GTGCCGCCGAAAGAACTAC	Anti-sense	CTGCTGCCACCATTACCTG	165 bp	Both
CK-8	Sense	GAGGCATCACCGCAGTTAC	Anti-sense	TTGCTTCGAGCCGTCTTCT	223 bp	Both
AQP-3	Sense	TGACCAGTTCATAGGCACAGC	Anti-sense	ACACGAAGACACCCGCAAT	296 bp	Traditional–PCR
AQP-3	Sense	CTCGTGAGCCCTGGATCAAGCT	Anti-sense	GTTGTCGGCGAAGTGCCAGATT	119 bp	q-PCR
AQP-5	Sense	GCCACCTTGTCGGAATCTAC	Anti-sense	CCAGTCCTCGTCAGGCTCATA	233 bp	Traditional–PCR
AQP-5	Sense	CACCCCCCACCTTCCCCAACCCT	Anti-sense	CTAAACACCAGCAGCCCCACGCA	169 bp	q-PCR
IL-6	Sense	GGAGACTTGCCTGGTGAA	Anti-sense	CTGAGGTGCCCATGCTAC	330 bp	Traditional–PCR
IL-6	Sense	GAGCCCACCGGGAACGAAAGAGA	Anti-sense	CAGCAGGCAACACCAGGAGCAGC	125 bp	q-PCR
IL-8	Sense	GCCAAGGAGTGCTAAAGA	Anti-sense	CCCTACAACAGACCCACA	329 bp	Traditional–PCR
IL-8	Sense	TACTCCAAACCTTTCCACCC	Anti-sense	AACTTCTCCACAACCCTCTG	158 bp	q-PCR
MCP-1	Sense	CAGCCAGATGCAATCAATGC	Anti-sense	GTGGTCCATGGAATCCTGAA	198 bp	Both
ICAM-1	Sense	GGAGCCAATTTCTCGTGC	Anti-sense	GGAGTCGTTGCCATAGGTG	267 bp	Traditional-PCR
ICAM-1	Sense	GATTGTCATCATCACTGTGGTAG	Anti-sense	GCCTGTTGTAGTCTGTATTTCTT	111 bp	q-PCR
GAPDH	Sense	TCCTCCACCTTTGACGCT	Anti-sense	TCTTCCTCTTGTGCTCTTGC	177 bp	Both

### Expression of epithelial cell markers by confocal microscope

The isolated AEII cells were cultured for 21 days and seeded onto glass coverslips at 5 × 10^4^ cells/mL in 24-well plates, incubated at 37°C for 12 h, fixed in 4% paraformaldehyde for 10 min, and then were permeabilized using 0.1% Triton X-100 followed by blockade using 2% BSA for 1 h. The cells were probed with rabbit anti-human pro-SP-C polyclonal antibody (Millipore, 1:1000 Temecula, CA), goat anti-human *α*ENaC polyclonal antibody (Santa Cruz Biotechnology, 1:500, Santa Cruz, CA), mouse anti-human cytokeratin-8 monoclonal antibody (Abcam, 1:100, Cambridge, MA), goat anti-human AQP-5 polyclonal antibody (Santa Cruz Biotechnology, 1:500), for overnight at 4°C. A positive control was performed using Hela cells based on the instruction of the use of AQP-5 antibody).

The cells were washed three times with PBS at 10-min interval and then incubated with mixed secondary antibody Cy3 AffiniPure Goat anti-Rabbit (1:200; EarthOx, San Francisco, CA), Cy3 AffiniPure Donkey anti-Goat (1:200, EarthOx), FITC AffiniPure Donkey anti-Goat (1:200, EarthOx), and FITC AffiniPure Goat anti-Mouse IgG (1:200, EarthOx) for 1 h at 37°C in dark. After washing three times, the coverslips were mounted, and analyzed using Confocal Scanning Microscopy (LCSM, Nikon C1Si, Tokyo, Japan).

### Expression of epithelial and mesenchymal cell markers by Western blots

Protein extraction from AEII at 21 days, and Western blotting was carried out as described previously (Cabrera-Benitez et al. 2012). The following antibodies were used: mouse anti-human E-cadherin antibody (BD Bioscience, 1:2000, San Jose, CA), goat anti-human pro-SP-B polyclonal antibody (Santa Cruz Biotechnology, 1:500), mouse anti-human vimentin (1:1000, BD), rabbit anti-human GAPDH (1:10000, BD), goat anti-mouse, donkey anti-goat, goat anti-rabbit-conjugated to horseradish peroxidase (1:5000, EarthOx).

### Functional test of AEII cells

The AEII cells were seeded in six-well plates at 2 × 10^5^ cells per well. After incubation overnight, the complete SAGM was replaced with serum-free SAGM and starved for 24 h. The cells were then stimulated for 24 h with TNF-*α* at 0, 0.1, 1, and 10 ng/mL. The total RNA was extracted for detection of gene expression of the inflammatory cytokine IL-6 (used for clinical trials of sepsis) and chemokines (IL-8 and MCP-1, the most common chemoattractants) and ICAM-1 (a costimulatory molecule to crosstalk with its ligands on leukocytes). The expression of the housekeeping gene GAPDH was used for loading control. The sequences of primers are listed in Table2. The protein concentrations of IL-6 and IL-8 were measured in culture supernatants by human-specific ELISAs (R&D Systems, Minneapolis, MN).

### Culture of human lung epithelial cells and fibroblast

Primary human small airway epithelial cells (SAEC, Lonza, and Walkersville, MD) were cultured in SAGM medium. Human bronchial epithelial cells (BEAS-2B) and lung adenocarcinoma epithelial cell line (A549 cell, ATCC, Manassas, VA) were cultured in RPMI 1640 medium supplemented with 10% FBS. Primary human lung fibroblasts (gift from Professor Xu, Guangzhou Institute of Respiratory Diseases, China) were cultured in DMEM supplemented with 10% FBS.

### Statistic analysis

Statistical analysis was performed with SPSS 12.0. Results are expressed as mean ± SE. Comparisons between two groups were made using ANOVA. *P* < 0.05 was considered statistically significant.

## Results

### Yield, purity, viability, and morphology

**Figure 1:**
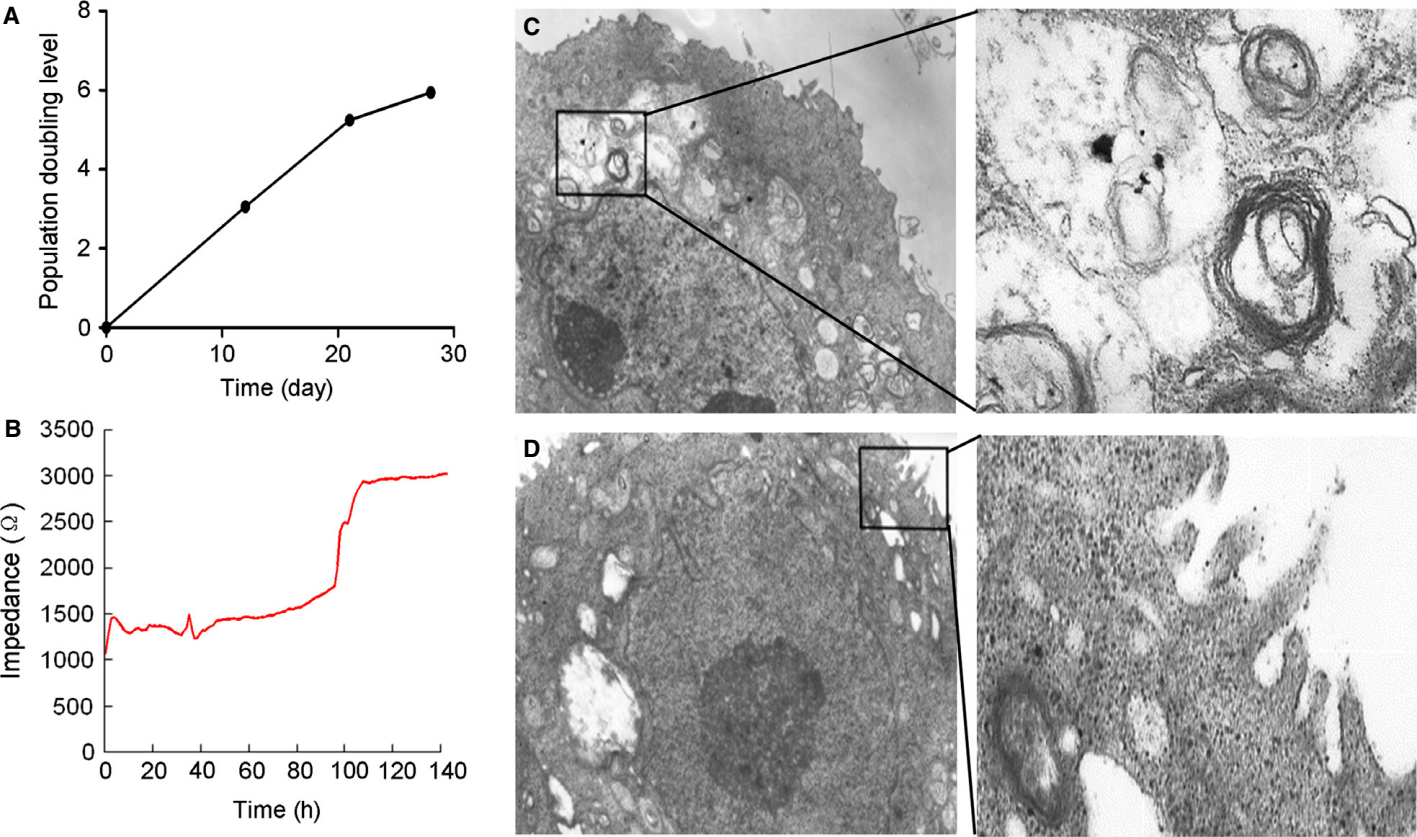
Cell growth curve, tight junction, and transmission electron microscopy structure of cultured primary human AEII cells. Left panels: (A) The AEII cells (1 × 10^4^/well) were seeded in SAGM containing 1% FBS using 24-well plates. Cell population doubling level was evaluated up to 28 days. (B) AEII cells (1 × 10^5^/well) were grown on electrodes standard 8-well array for measurement of the difference of transepithelial resistance values between the electrodes with and without cell coverage. The resistance value remained stable when formation and maturation of a confluent cell barrier were reached after 120 h of culture. Right panels: Isolated primary human AEII cells were cultured for 21 days and undertaken electron microscopy. The box represents lamellar bodies (C) at high power (8900×), and microvilli at high power (D, 6610×), respectively.

Our isolation process resulted in yielding approximately 2–7 × 10^5^ human AEII cells per gram peripheral lung tissue at 60 h of the cell culture. The viability was 95 ± 2% averaged from 28 lungs over 60 h. There was no difference in cell growth among the use of different culture medium but the morphology of AEII cells appeared to be optimal under the condition of SAGM containing 1% FBS but not under that of DMEM containing 1% or 10% FBS or SAGM containing 10% FBS (data not shown). The population doubling level was about 6-fold at day 28 (Fig.1A). The cells formed tight junction after reaching confluence as reflected by a constant reading of ECIS (Fig.1B). The typical lamellar bodies and microvilli were seen in the AEII cells under the transmission electron microscopy (Fig.1C and D).

### Molecular characteristics of AEII cells

**Figure 2:**
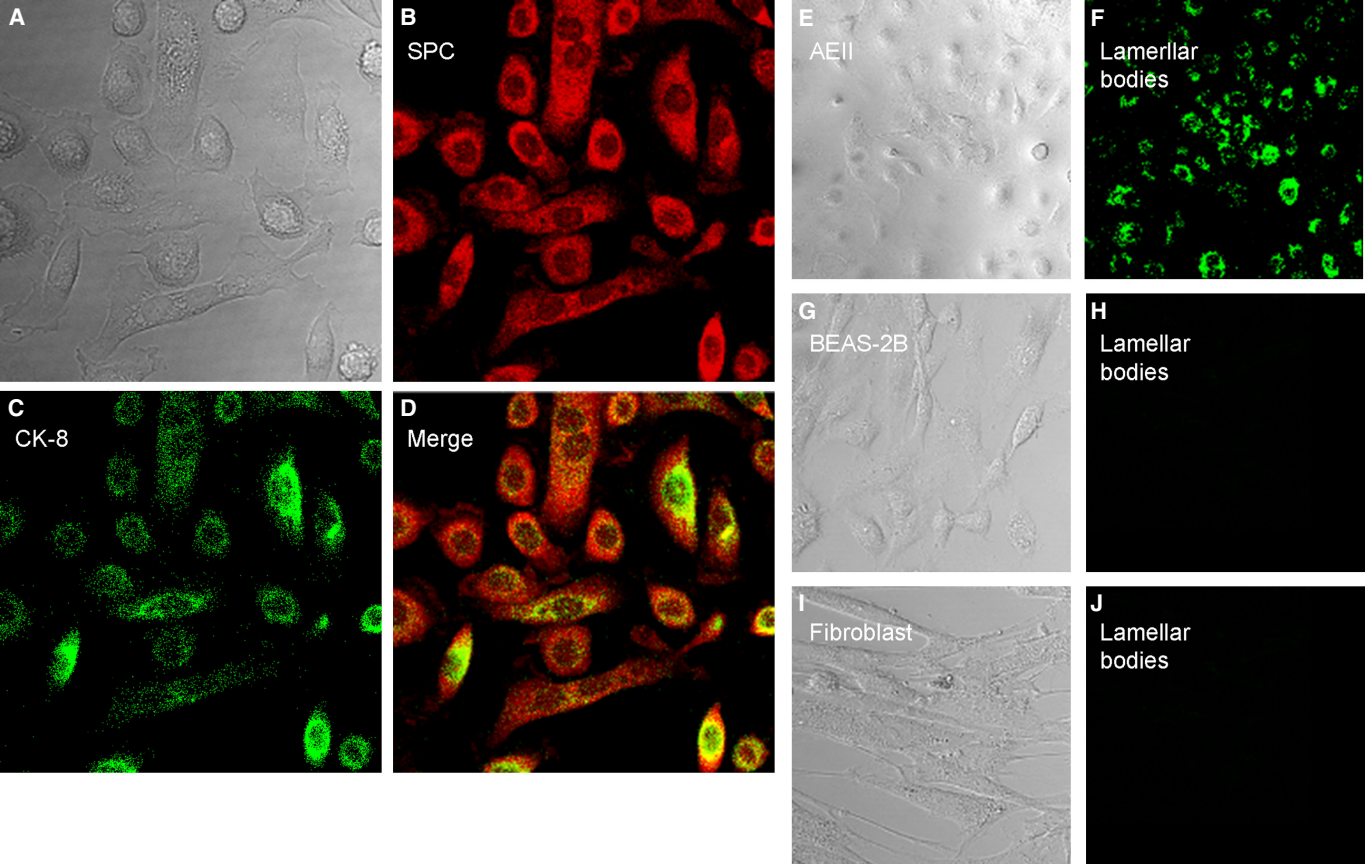
Expression of AEII markers and lamellar bodies. (A–D) The isolated AEII cells were cultured for 21 days (A, DIC) and were probed with a rabbit anti-human pro-SPC (SPC) polyclonal antibody (B, red) and a mouse anti-human cytokeratin-8 (CK-8) monoclonal antibody (C, green), followed by incubation with Cy3 Goat anti-Rabbit and FITC Goat anti-Mouse IgG. The SPC and CK-8 were colocalized (D). (E–J) The AEII cells were incubated with LysoTracker® Green DND-26 (E and F) for staining of lamellar bodies, and analyzed under confocal scanning. Green DND-26 staining was negative in human bronchial lung epithelial BEAS-2B cells (G and H) or human lung fibroblasts (I and J).

The AEII cells were characterized by the expression of proteins specific for AEII cells including pro-SP-C (Fig.2B) and cytokeratin-8 (Fig.2C). Lamellar bodies as specific feature of AEII cells (Williams 1977; Witherden et al. 2004) were positively identified by DND-26 staining in the cytoplasm (Fig.2E and F). The lamellar bodies were not found in the human bronchial lung epithelial BEAS-2B cells (Fig.2G and H) and the human lung fibroblasts examined (Fig.2I and J).

**Figure 3:**
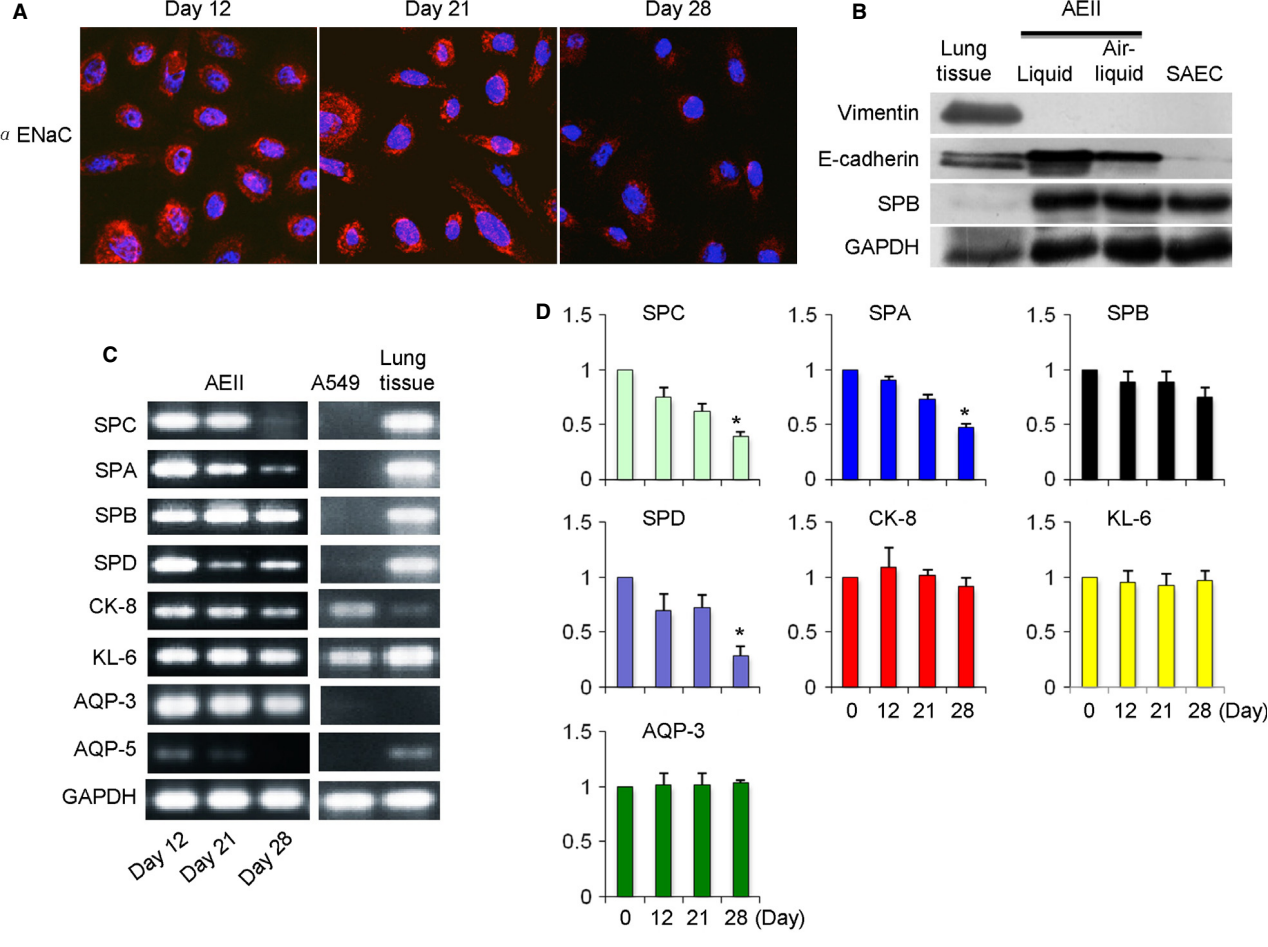
Expression of AEII cell and AEI markers. (A) AEII cells were cultured and expression of αENaC (red) was detected at days 12, 20, and 28 after isolation, while nuclear was stained by DAPI (blue). (B) Expression of the tight junction protein E-cadherin, surfactant protein B (SPB), and the mesenchymal marker vimentin at day 21 after AEII isolation in liquid or air–liquid culture conditions, respectively. Lung tissue from which primary AEII cells were isolated served as a positive control for vimentin. Primary human small airway epithelial cells (SAEC) served as a negative control for vimentin. (C) Gene expression of surfactant proteins (SPC, SPA, SPB, and SPD), cytokeratin-8 (CK-8), KL-6, AQP-3 (AEII cell marker), and AQP-5 (AEI cell marker) using traditional PCR methods at days 12, 20, and 28 after isolation of the primary AEII cells (*N *= 28), and in A549 cells and lung tissue from which primary AEII cells were isolated. (D) Gene expression of SPC, SPA, SPB, SPD, CK-8, KL-6 and AQP-3 by RT-qPCR. *P < 0.05 versus D0, respectively.

**Figure 4:**
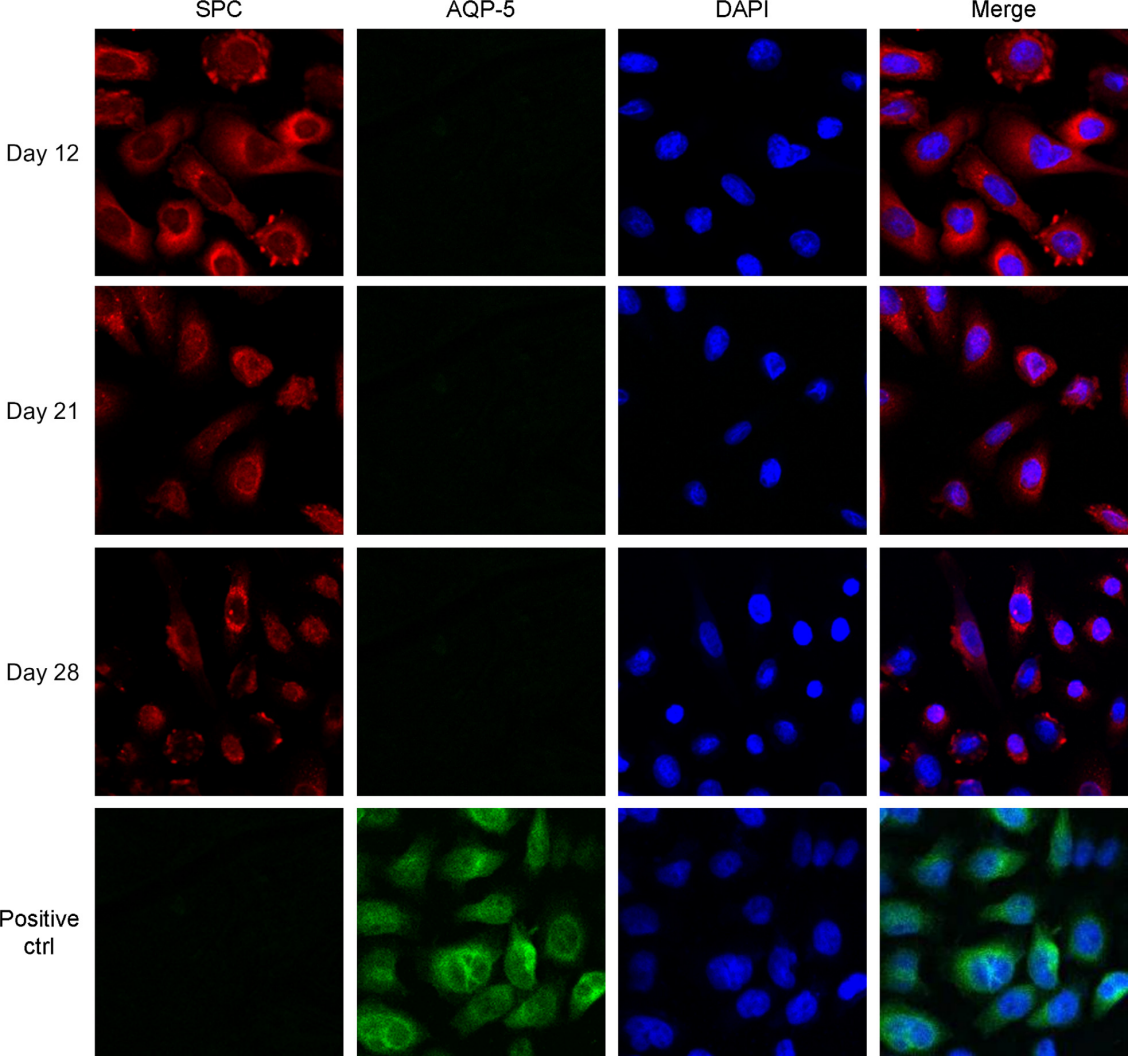
Expression of SPC and absence of expression of AQP-5 in AEII cell. Cultured AEII cells or Hela cells (5 × 10^4^ cells/mL) were seeded for 12 h, fixed in 4% paraformaldehyde, and then were permeabilized using 0.1% Triton X-100 followed by blockade using 2% BSA. The cells were immunostained by using rabbit anti-human pro-SPC polyclonal antibody and goat anti-human AQP-5 polyclonal antibody over night at 4°C. After washing, the cells were incubated with appropriate secondary antibodies for 1 h at 37°C in dark. The coverslips were mounted after washing, and analyzed under a confocal microscope.

To monitor the phenotype of AEII cells after isolation, the cells were cultured in SAGM containing 1% FBS for up to 28 days. The expression of *α*ENaC (Fig.3A), the tight junction protein E-cadherin, and the AEII protein SPB (Fig.3B) and SPC (Fig.4), but not the mesenchymal protein vimentin (Fig.3B) and the AEI protein AQP-5 (Fig.4), was detectable. There was no difference in the expression of E-cadherin and SPB at day 21 between the liquid and air–liquid culture conditions (Fig.3B). The whole lung tissue from which the AEII cells were isolated showed strong expression of vimentin (Fig.3B).

Moreover, the gene expression of surfactant proteins, CK-8, KL-6, and AQP-3 as AEII markers (Driscoll et al. 1995; Armstrong et al. 2004; Bove et al. 2010) was observed at 12 (passage 1), maintained at day 21, and decreased at day 28 (Fig.3C). There was little expression of AQP-5 at day 12 and it disappeared thereafter (Fig.3C). The AEII gene profile was further confirmed using the real-time qPCT methods (Fig.3D). In contrast, the human adenocarcinomic epithelial A549 cells did not show any expression of the surfactant proteins and AQP-3 (Fig.3C).

### Functional response of AEII cells upon stimulation

**Figure 5:**
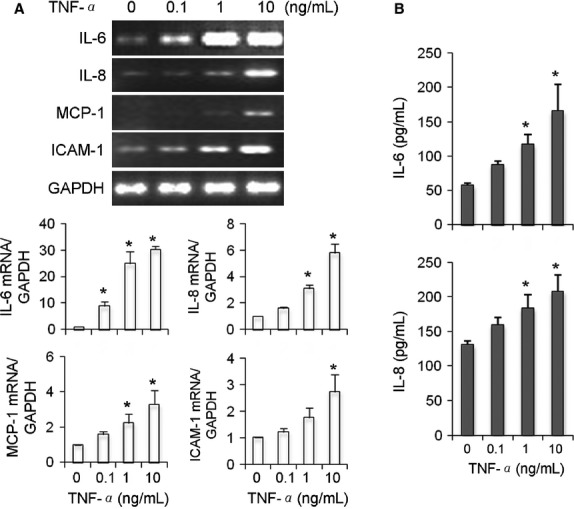
Gene expression IL-6, IL-8, MCP-1, and ICAM-1 in response to TNF-*α* stimulation. Isolated primary human AEII cells were cultured for 21 days and stimulated with TNF-*α* for 24 h. A. Total RNA was extracted from cell lysates and probed for mRNA expression of IL-6, IL-8, MCP-1 and ICAM-1. B. Protein concentrations of IL-6 and IL-8 were measured in culture supernatants. *P < 0.05 versus 0 (PBS vehicle control).

To examine functionality of the primary AEII cells, the cells were stimulated with different doses of TNF-*α* for 24 h. There was a dose-dependent increase in the gene expression of IL-6, IL-8, MCP-1, and ICAM-1, as well as the protein concentrations of IL-6 and IL-8 in culture supernatants in response to the stimulation with TNF-*α* (Fig.5).

## Discussion

There are a couple of highlights in the present method study with respect to the isolation and maintenance of primary human AEII cells: (1) The high yielding was achieved by combined application of trypsin with elastase digestion that preserved the lung tissue during harvest; (2) The high purity was obtained by separation of macrophages and fibroblasts from epithelial cells at an optimized time using adhesion medium antibodies for negative selection; and (3) The prolonged maintenance of AEII phenotype was accomplished by optimization of the components of culture medium containing an appropriate FBS concentration.

AEII cells, as tissue stem cells, have very important functions. It is necessary to establish primary culture of human AEII cells for our understanding of the cellular and molecular mechanisms under pathological conditions of the lung diseases such as the lethal ARDS and ventilator-induced lung injury. Since Dobbs (Dobbs et al. 1986) first published a method of isolation and culture of adult rat ATII cells, a number of studies have been carried out to isolate AEII cells in humans (Fuchs et al. 2003; Bove et al. 2010) and in rodents (Bates et al. 2002; Witherden et al. 2004; Lazrak et al. 2012). The studies have shown that the isolated human AEII cells exert function in vitro (Wang et al. 2007, 2011; Ballard et al.^2010^; Qian et al. 2013; Bove et al. 2014). However, several major concerns have arose including rapid loss of cell function, especially loss of surfactant protein expression (Dobbs 1990; Bates et al. 2002; Witherden et al. 2004) and cell differentiated into ATI phenotype within a few days (Bates et al. 2002; Bove et al. 2010).

Maintenance of cell characteristics depends on not only the source of tissue but also the methods of isolation and the components of culture medium. In this study, we modified the protocols from several previous studies for AEII cell isolation, characterization, and culture. First, we used the normal peripheral lung tissue from patients undergoing lung resection as our tissue source. Second, the combined application of trypsin with elastase was able to decrease the dosage of trypsin and thus may have helped reduce tissue injury and improved harvest (Waymouth 1974). Third, we believe that the removal of fibroblasts is a crucial step during the isolation and purification procedure (Komiyama et al. 2003; Kaushik et al. 2008). Finally, the SAGM containing 1%FBS was an important recipe in maintaining the biological characteristics of human AEII cells and preventing their differentiation into AEI cells.

There are several kinds of mediums including low protein hybridoma media, SAGM, and DMEM being used (Pechkovsky et al. 2000; Bur et al. 2006; Thorley et al. 2007; Kosmider et al. 2010) for human AEII cell culture. SAGM contains epinephrine, hydrocortisone, and retinoic acid, which appears to be important for inhibition of differentiation (Rippon et al. 2004). The epidermal growth factor in SAGM can promote cell proliferation in mammalian cells (Worster et al. 2012). We demonstrate that SAGM supplemented with 1% FBS but not 10% FBS was able to maintain the AEII phenotypes, but the other kinds of medium containing different concentration of FBS failed to maintain the AEII cell phenotype. Although the AEII cell population doubling was somewhat slow, the phenotype of AEII cells appeared to last longer (i.e., over 20 days) as compared to previous studies (Bates et al. 2002; Witherden et al. 2004; Bove et al. 2010). This time frame is usually long enough for common mechanistic studies in vitro following insults such as mechanical stretch, hypoxic exposure and acid challenge (Ishizuka et al. 2001; Bouvry et al. 2006; Cabrera-Benitez et al. 2012).

The primary human AEII cells were characterized by the expression of pro-SP-C (Bove et al. 2010), cytokeratin-8 (Driscoll et al. 1995), *α*ENaC (Lazrak et al. 2012), and lamellar bodies (Haller et al. 1998; Fang et al. 2006; Wemhoner et al. 2011). A technical challenge of primary AEII cell culture was the rapid loss of expression of surfactant proteins (Dobbs 1990; Witherden et al. 2004). Our AEII phenotype was evidenced by the sustained expression of surfactant proteins (Ballard et al. 2010; Wang et al. 2011; Qian et al. 2013), epithelial markers (KL-6, CK-8) (Driscoll et al. 1995; Hermans and Bernard 1999), and AEII cell differentiation marker of AQP-3 while lacking AQP-5 of an AEI cell marker (Armstrong et al. 2004). Finally, the primary AEII cells also showed excellent biological function by upregulation of cytokines, chemokine, and adhesion molecule at gene and protein levels in response to stimulation with TNF-*α*. This provides an important tool to study inflammatory responses involved in AEII cells.

In conclusions, we have established techniques for human lung tissue harvest and AEII cell isolation that allow for the primary culture of these cells over an extended period of time. The technique provides a reliable tool to examine the cellular and molecular mechanisms of human AEII cells in the context of acute and chronic lung diseases associated with alveoli.

## Conflict of Interest

None declared.
